# Menthol, a unique urinary volatile compound, is associated with chronic inflammation in interstitial cystitis

**DOI:** 10.1038/s41598-018-29085-3

**Published:** 2018-07-18

**Authors:** Muhammad Shahid, Min Young Lee, Austin Yeon, Eunho Cho, Vikram Sairam, Luis Valdiviez, Sungyong You, Jayoung Kim

**Affiliations:** 1 Departments of Surgery and Biomedical Sciences, Cedars-Sinai Medical Center, Los Angeles, CA USA; 2 Institute for Systems Biology, Seattle, WA USA; 3 University of California Los Angeles, Los Angeles, CA USA; 4 West Coast Metabolomics Center, UC Davis, Davis, CA 95616 USA; 5 Samuel Oschin Comprehensive Cancer Institute, Cedars-Sinai Medical Center, Los Angeles, CA USA; 6 Department of Urology, Ga Cheon University College of Medicine, Incheon, South Korea

## Abstract

Chronic inflammation is a potential systemic risk factor for many bladder dysfunctions, including interstitial cystitis (IC). However, the underlying mechanism through which a healthy bladder protects itself from inflammatory triggers remains unknown. In this study, we identified odor compounds in urine obtained from IC patients and healthy controls. Using comprehensive solid-phase microextraction-gas chromatography-time-of-flight-mass spectrometry (SPME-GC-TOF-MS) profiling and bioinformatics, we found that levels of urinary volatile metabolites, such as menthol, were significantly reduced in IC patients, compared to healthy controls. In an attempt to understand the mechanistic meaning of our volatile metabolites data and the role of menthol in the immune system, we performed two independent experiments: (a) cytokine profiling, and (b) DNA microarray. Our findings suggest that lipopolysaccharide (LPS)-stimulated inflammatory events, such as the production and secretion of inflammatory cytokines (e.g., TNF-α, IL-6, and IL-1β) and the activation of NF-κB and associated proteins within a large signaling network (e.g., Akt, TLR1, TNFAIP3, and NF-κB), are suppressed by the presence of menthol. These findings broaden our knowledge on the role of urinary menthol in suppressing inflammatory events and provide potential new strategies for alleviating both the odor and inflammation associated with IC.

## Introduction

Interstitial cystitis (IC) is a clinical condition that presents itself as sensory hypersensitivity of unknown cause and is characterized by frequent urination, bladder discomfort, and pelvic pain^1^. IC occurs in both women and men over a broad age range and across ethnic/racial groups^2^. In the United States, more than 3–8 million women and 1–4 million men are diagnosed with IC annually^3^. The prevalence estimates of IC vary substantially because of differences in source populations and case ascertainment^4^. Current diagnostic techniques include cystoscopy, potassium sensitivity tests, hydrodistension *et al*. However, these procedures are not only invasive, painful, and inconvenient, but also extremely costly, complicated, and minimally informative. In addition to these complications with diagnosis, the lack of consensus regarding the cause of IC has resulted in difficulties determining effective and specific therapies.

Although there has been immense progress in the fields of genomics and proteomics, further research into the biological end points of human diseases is needed for improved disease diagnosis, prognosis, and therapeutic development. In recent years, metabolomic profiling, also known as metabolomics, has been viewed as a promising technique in disease diagnosis. Metabolomics focuses on utilizing and analyzing metabolites and biomarkers as signals for cellular states. These biological biomarkers have been used to understand the metabolic changes that occur over time in a variety of diseases^5^. In particular, clinical samples, such as tissues and biofluids (e.g. serum, plasma, urine, and saliva), have proven to be valuable sources for diagnostic purposes. For instance, human plasma proteins originate from a variety of cells and various medical studies have shown that these proteins reflect human physiological and pathological states. Therefore, they can potentially be utilized to increase diagnostic efficiency and prognostic efficacy^6^. Other biological fluids have been quantitatively determined in regards to their metabolic composition through procedures such as gas chromatography, high-pressure liquid chromatography, mass spectrometry.

Urine contains a multitude of water-soluble waste products filtered through the kidneys and eliminated from the body via micturition. It contains many metabolites, such as urea (from amino acid metabolism), inorganic salts (chloride, sodium, and potassium), creatinine, ammonia, organic acids, water-soluble toxins, and urobilin. While this complexity can make urine analysis difficult, the potential information that can result will be very beneficial, and progress in the field has been promising. Additionally, collection of urine is simpler and provides a relatively larger volume of sample compared to other biological fluids.

Odor consists of various volatile organic chemical compounds (VOCs), which can be identified through mass spectrometry. Compared to other organic compounds, VOCs generally have a lower molecular weight and higher vapor pressure. Many prior studies have applied VOCs into cancer research. For cancer detection, there have been several studies on using gas chromatography-mass spectrometry (GC-MS) to detect certain odor compounds in skin, tissue, breath, feces, and bodily fluids, such as sweat and urine^7–9^. VOCs can also be used to assist in the diagnosis of lung and prostate cancer^10^. Electronic noses capable of detecting odor signatures have been developed and successfully applied in discriminating prostate cancer patients from healthy controls^9,11^. In terms of IC, perturbed VOCs may underlie the commonly reported changes in urine odor. IC is known to negatively impact overall quality of life through its effects on urinary odor and leakage; many patients often report foul smelling urine^12^. Given our previous findings that IC patients may have a distinct metabolism^13,14^, we hypothesized that urine from IC patients might contain a distinguishing VOC signature that is reflective of disease conditions.

In our present volatile metabolomics study, we tested the hypothesis that urinary VOCs differ between IC patients and healthy controls. Using urinary samples from the urine headspace of IC patients and healthy controls. We extracted VOCs via solid-phase micro-extraction and analyzed them using GC-MS. The aim of this study was to identify IC-associated VOCs and further examine their biological meaning in the bladder epithelium. From our comprehensive and unbiased metabolomics analysis, we found menthol to be a novel compound involved in IC-associated inflammation. We discovered that urinary menthol decreased in IC patients and that these reduced levels are potentially linked to the chronic inflammation commonly observed in IC.

## Materials and Methods

### Cell Line

The mouse macrophage cell line, RAW 264.7, was obtained from Sigma Cells (St. Louis, MO, USA) and was cultured in Dulbecco’s Modified Eagle Medium (DMEM) supplemented with 10% fetal bovine serum (FBS). The cells were kept in humidified incubators with 5% CO_2_ at 37 °C. The medium was replaced every day and the cells were passed every two to three days to maintain logarithmic growth.

### Reagents

Menthol and bacterial lipopolysaccharide (LPS) (*Escherichia coli*, 0111: B4) were purchased from Sigma (USA). Mass spectrometry grade reagents (column, buffer *et al*.) were all purchased from Sigma (USA). The Mouse Proteome Profiler Array was purchased from R&D Systems (USA). The antibodies used were CCL3 (ab25128, Abcam, USA), IL-6 (12912; Cell Signaling Technology, USA), TNF-α (11948S; Cell Signaling Technology, USA), p-NF-κB (3033; Cell Signaling Technology, USA), NF-κB (8242; Cell Signaling Technology, USA), p-Akt (4051; Cell Signaling Technology, USA), Akt (9272; Cell Signaling Technology, USA), TLR1(2209; Cell Signaling Technology, USA), TNFAIP3 (5630; Cell Signaling Technology, USA), IFIT1 (14769; Cell Signaling Technology, USA), viperin (13996; Cell Signaling Technology, USA), IL-1β (AF-401-NA, R&D Systems, USA), and β-actin (A1978; Sigma-Aldrich, USA). HRP-conjugated secondary antibodies were obtained from Cell Signaling Technologies (7074, 7076; USA).

### Ethics Statements

The ethics committee at Inha University Hospital (Incheon, South Korea) approved this study. Written informed consent was obtained from all subjects. The Institutional Review Board of Inha University Hospital approved collection, curation, and analysis of all samples (IRB #10-0751)^13,14^. All methods were performed in accordance with the relevant guidelines and regulations.

### Subjects and Urine Specimen Collection

Patients and healthy control subjects were recruited from an outpatient urology clinic at Inha University Hospital. All subjects were Asian females. Subjects were instructed to avoid tobacco, nicotine, chemical compounds, alcohol, herbal foods, caffeine, and medication 24 hrs before their urine collection. Recruitment was conducted following the National Institute of Diabetes and Digestive and Kidney Diseases (NIDDK) guidelines. Workup included symptom assessment, cystoscopic evaluation, physical examination, urodynamics, and/or urine culture. Patients with a history of other diseases, including cancer, chronic inflammation, or diabetes, were excluded.

To minimize possible contamination with vaginal, rectal, or urethral cells, first morning urine specimens were obtained using clean catch methods in a sterile environment. The de-identified specimens were sent to laboratory and centrifuged for 10 mins to remove cell debris. Urine supernatants were then processed into individual aliquots and stored in 15 ml tubes at −80 °C until further analysis.

### Availability of data and materials

All the data supporting the findings here is contained within the manuscript.

## Volatile Metabolomics

### Sample preparation

Metabolomics analysis was performed using urine samples obtained from IC diagnosed (n = 10) and healthy age-matched controls (n = 10). Urine samples were prepared in triplicates in 20 ml amber headspace vials with magnetic screw caps and silicone/PTFE septa. Urine samples (10 ml) were added to a vial containing 2.5 g of sodium chloride that had been dried at 100–150 °C for at least 2 hrs prior to weighing. The solution was capped, vortexed, and loaded onto an autosampler tray. No more than 18 samples were prepared at a time; this was done to minimize the length of time the last sample in the batch sat at ambient laboratory temperature prior to extraction and analysis. Quality control experiments were performed as described in a previous paper^15^.

### Solid-phase microextraction (SPME) and GC-TOF-MS analysis

A LECO Pegasus III Time-of-Flight Mass Spectrometer (LECO, St. Joseph, MI, USA) equipped with an Agilent 6890 Gas Chromatograph (Agilent Technologies, Santa Clara, CA, USA) was used for analysis. ChromaTOF (ver. 4.50.8.0, LECO) was used for raw data processing, including automatic peak detection and deconvolution, as described in a previous paper^15^.

### Urine GC-MS data pre-processing

We performed data cleaning and pre-processing using Excel and R Studio^15^. A two-way analysis of variance comparing the first and last four samples did not show any differences in false discovery rate (FDR) corrected significance levels for peak abundances.

### Differential expression analysis for volatile metabolite profiles

To identify differentially expressed metabolites between the urines of IC patients and controls, we applied the integrative hypothesis testing method^16^. The t-test, log2-median-ratio test, and Wilcoxon rank sum test were also performed. For the t-test and log2-median-ratio test, an empirical distribution of the null hypothesis (the means of the metabolite intensity levels are not different) was estimated using random permutations of the samples. For each metabolite, an p-value was computed by performing a two-tailed test on the empirical distributions. The three p-values were combined using Stouffer’s method to compute the adjusted p-values. The FDR was computed from the adjusted p-value using Storey’s method^17^. We identified 12 metabolites with a FDR < 0.1.

### Western blot analysis

Cells were lysed with a RIPA buffer (20 mM Tris, 150 mM NaCl, 1% Nonidet, P-40, 0.1 mM EDTA) (Pierce, ThermoFisher) that was supplemented with a phosphatase inhibitor cocktail (ThermoFisher). The protein concentration of each sample was measured using the Bradford Protein Assay Kit, according to the manufacturer’s protocol (Pierce, ThermoFisher). Equal amounts of extracts were separated by SDS-PAGE and transferred onto a PVDF membrane. The membranes were then blocked with 5% bovine serum albumin or 5% nonfat milk in tris-buffered saline with tween 20 (TBST) [2.42 g/L Tris-HCl, 8 g/L NaCl, and 1 mL/L Tween 20 (pH 7.6)] and incubated overnight at 4 °C with specific primary antibodies in TBST. Following this first incubation, the membranes were washed and incubated again with horseradish peroxidase-conjugated secondary antibodies. β-actin was used as an internal control.

### Cytokine array

Cell lysates and conditioned media were collected and analyzed using a cytokine array from R&D Systems (USA). They were then diluted and mixed with a cocktail of biotinylated detection antibodies. The sample/antibody mixture was then incubated with the mouse cytokine array membrane. Any cytokine or detection antibody complex was bound to its cognate immobilized capture-antibody on the membrane. Following a wash to remove unbound material, streptavidin-HRP and chemiluminescent detection reagents were added sequentially. Light was produced at each spot in proportion to the amount of cytokine bound. For data quantification ImageJ was used.

### RNA preparation for DNA microarray analysis

Total RNA was extracted from RAW 264.7 macrophage cells that were treated with LPS and/or menthol, using a Qiagen RNEasy Mini Kit (Qiagen Inc., Valencia, CA, USA). The RNA concentration of the samples and quality controls was measured using the Bioanalyzer 2100 and Nanodrop 8000a (ThermoScientific, Willmington, DE, USA).

### Microarrays and data analysis

Total RNA (200 ng) was transcribed to double-stranded cRNA using the MessageAmp Primer RNA Amplification Kit (Life Technologies, Carlsbad, CA, USA) with an oligo(dT) primer, according to the manufacturer’s instructions. After fragmentation, 11 µg of biotin-labeled cRNA was hybridized for 16 hrs at 45 °C on the Affymetrix Mouse Genome 430 Plus 2.0 Array (Affymetrix, Santa Clara, CA, USA). GeneChips were then washed and stained in the Affymetrix Fluidics Station 450 and scanned using the Affymetrix GeneChip Scanner 3000 G7 (Affymetrix). Quality control was performed with the Affymetrix Expression Console software (Affymetrix version 1.3). The raw data was normalized using the gcrma package (version 2.10.0) in R 2.6.1. The log2 GC-RMA signals were then exported and used for differential expression analysis. Both the CEL files and normalized data discussed here are deposited and available at Gene Expression Omnibus (http://www.ncbi.nlm.nih.gov/geo/) under the accession number GSE98933.

To identify differentially expressed genes (DEGs), we used a two-tailed Welch’s t-test. DEGs were identified as genes with a p-value < 0.05 and fold-change ≥1.5. In order to reduce unreliable detection and false positives, probe sets with average expression levels higher than the average of all probe sets in the data were also considered for further analysis. To identify biological processes affected by LPS or menthol, we performed functional enrichment analysis of Gene Ontology Biological Processes (GOBPs) using DAVID^18^. Those with p-values < 0.05 and DEGs ≥3 were selected as significantly represented GOBPs.

### Statistical analysis

The mean of more than three replicates was used as the average. For simple comparisons, p-values were calculated using a standard unpaired Student’s t-test. Statistical significance was considered as p < 0.05.

## Results

### Volatile metabolomics profiling revealed that menthol levels are significantly reduced in the urine specimens of IC patients

We sought to determine the VOC composition of urine in IC and healthy patients by performing volatile metabolite profiling using SPME-GC-TOF-MS. We used fatty acid methyl esters (FAMEs) as internal standards for quality control (including in injections) and for retention index corrections. The method used for untargeted profiling was based on the method developed by Robinson^19^, with some added modifications (see materials and methods). The ChromaTOF software was used for automatic peak detection and deconvolution of the raw data. After data cleaning and pre-processing, a total of 113 peaks were identified. Quality assessment of these quantification results was done after quantile normalization^20^ (Supplementary Fig. 1). Peak intensities were summed for all identified metabolites (mTIC). Each peak was then normalized to the sample’s total volatile metabolome.

**Figure 1:**
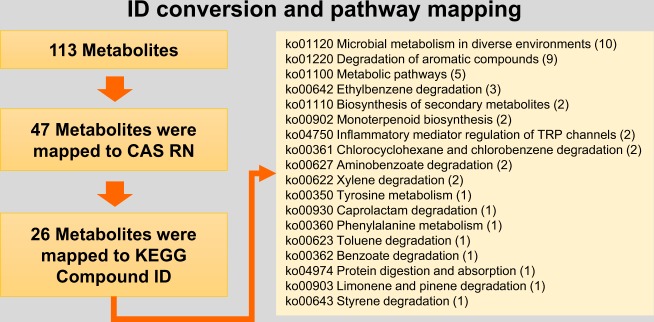
The analysis workflow of volatile metabolome identification, conversion to KEGG IDs, and pathway mapping.

We then investigated the association of these peaks with known metabolic pathways. To do this, we first selected 47 peaks that were annotated with a CAS registry number and then identified 26 peaks that can be mapped to at least one Kyoto Encyclopedia of Genes and Genomes (KEGG) compound ID. Using the DAVID software^18^, we found that 26 metabolites were associated with 26 pathways (Fig. 1). We next performed differential analysis to identify which metabolites were significantly altered between IC patients and healthy controls. The analysis included 76 peaks, with quantification of more than half of the samples in each condition. This resulted in 12 peaks that were identified with a FDR <0.1 (Table 1). The FDR was calculated using the integrated hypothesis testing method (see materials and methods)^16^. Among them, menthol (CAS RN: 89-78-1) was identified to be significantly different, with a FDR of 0.024 and log2 fold-change of −0.1.467. We observed only authentic VOCs, not chemicals or metabolites that could come from degradation processes.

**Table 1:** Differentially expressed metabolites in urine specimens obtained from IC patients compared to health controls (FDR < 0.1).

Name	CAS Registry Number	KEGG	Retention Time	FDR	FC	SD
Benzaldehyde,3,5-dimethyl-	5779-95-3	NA	540.902	0.005	5.246	3.261
**Cyclohexanol, 5-methyl-2-(1-methylthyl)-, (1a,2a,5a)-(n)-**	**89-78-1**	**C00400**	**520.734**	**0.024**	**−1.467**	**1.381**
t-Butyl ethyl ether2	1634-04-4	C11344	228.028	0.026	−0.539	0.597
yy054	NA	NA	526.908	0.027	−1.092	0.767
yy088	NA	NA	773.515	0.046	0.332	0.278
yy003	NA	NA	193.042	0.049	−0.574	1.109
Benzene, (1-methyl-1-butenyl)-	53172-84-2	NA	527.026	0.053	−0.974	0.737
yy032	NA	NA	455.642	0.063	0.383	0.372
2-Pentanone	107-87-9	C01949	295.78	0.074	−0.718	1.006
yy082	NA	NA	708.541	0.085	0.429	0.671
Benzene, (2-isothiocyanatoethyl)-	2257-09-2	NA	643.92	0.092	1.445	1.298
yy010	NA	NA	366.443	0.096	0.770	0.995

Menthol (KEGG compound ID: C00400) was expressed less in IC patients, compared to controls. The CAS registry numbers of the identified metabolites were mapped to their KEGG compound IDs. The FDR indicates the false discovery rate, which was computed using Storey’s method. FC is log2 fold change between IC and control. SD represents standard deviation. Positive and negative FC values indicate up- and downregulation of the metabolite in IC urine, compared to control (NA meaning not available).

Given our results demonstrating reduced menthol levels in the urine of IC patients and prior knowledge from literature, we speculated that menthol may be influencing bladder health. We hypothesized that the urine of IC patients contains reduced levels of anti-inflammatory metabolites, particularly menthol, which leads to an increase in IC-associated cytokines. To test this hypothesis, we sought to evaluate whether the anti-inflammatory effects of menthol could suppress LPS-induced inflammatory events in immune cells. We decided to use two independent approaches: (i) mesoscale cytokine profiling, and (ii) gene expression microarray analysis.

In order to characterize the effects of menthol on macrophages, comprehensive microarray analysis was conducted on RAW 264.7 cells under various conditions. Cells were treated with menthol or control vehicle for 1 hr. The macrophages were then stimulated with LPS treatment (100 ng/ml) for the following 6 hrs. Three groups of gene expression profiles were defined: control vs menthol (C vs M), control vs LPS (C vs LPS), and menthol vs LPS (M vs LPS).

### Cytokine profiling revealed that menthol downregulated the LPS-induced production of inflammatory cytokines in RAW 264.7 macrophages

**Figure 2:**
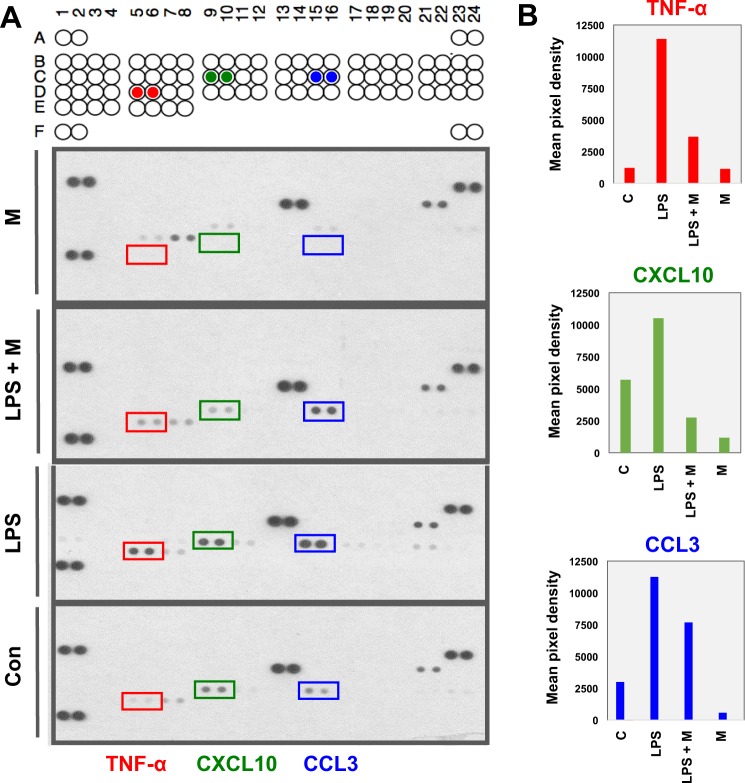
Reduced production of LPS-induced inflammatory cytokines by menthol in RAW 264.7 cells (cell lysates) (**A**) Inflammatory cytokine array analysis of CCL3, CXCL10 and TNF-α. The expression of these inflammatory cytokines is highly induced by LPS, but downregulated by menthol. (**B**) Quantification of array band intensity of CCL3, CXCL10 and TNF-α with ImageJ-analysis software.

An inflammatory cytokine array was used to identify the specific cytokines that were produced and secreted into the surrounding medium by the RAW 264.7 cells. To determine whether the presence of menthol affects the production and release of cytokines, RAW 264.7 cells were pretreated with menthol (500 µmol/ml) or control for 1 hr and then induced with LPS (100 ng/ml) for 6 hrs. The expression of each inflammatory cytokine was subsequently measured. The cytokine profiling data showed that a series of cytokines, including C-C motif chemokine ligand 3 (CCL3), C-X-C motif chemokine ligand 10 (CXCL10), and tumor necrosis factor alpha (TNF-α), were induced by LPS (Fig. 2A, LPS condition).

Compared to treatment with LPS alone, the addition of menthol significantly downregulated the production and secretion of these cytokines (Fig. 2A, LPS+M compared to LPS). ImageJ analysis software was used to measure of the values of the scan dots, according to their intensity on the cytokine array panel (Fig. 2B). These results suggested that menthol is involved in downregulating the production of LPS-induced inflammatory cytokines.

**Figure 3:**
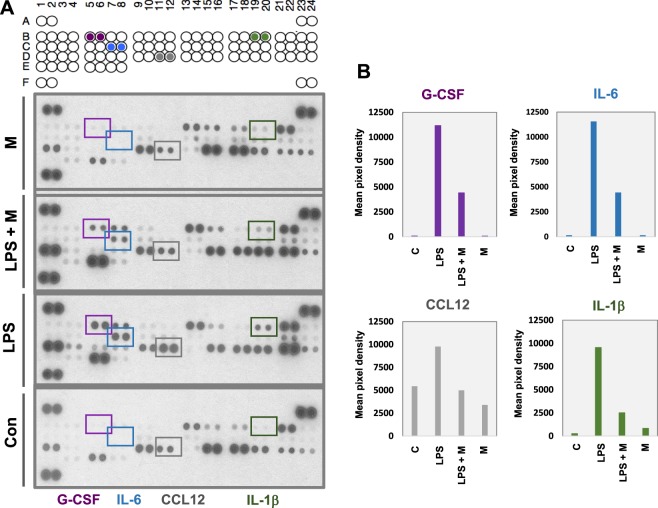
Decreased secretion of LPS-induced inflammatory cytokines by menthol in RAW 264.7 cells (conditioned media) (**A**) Inflammatory cytokine array analysis of IL-1β, IL-6, CCL5, CCL12 and G-CSF. The expression of these inflammatory cytokines is highly induced by LPS, but downregulated by menthol. (**B**) Quantification of array band intensity of IL-1β, IL-6, IL-1 and G-CSF with imageJ-analysis software.

We also carried out the inflammatory cytokine array to identify which cytokines were secreted into the RAW 264.7 cell culture media. We detected increased secretion of inflammatory cytokines, including interleukin 1β (IL-1β), interleukin 6 (IL-6), C-C motif chemokine ligand 5 (CCL5), C-C motif chemokine ligand 12 (CCL12), and granulocyte colony stimulating factor (G-CSF), in the LPS-treated condition (Fig. 3A). We also found that pretreatment with menthol significantly downregulated the secretion of these cytokines, compared to LPS alone (Fig. 3A, LPS+M compared to LPS). The dot intensities of each inflammatory cytokine were quantified using ImageJ software, as described in the Methods (Fig. 3B).

**Figure 4:**
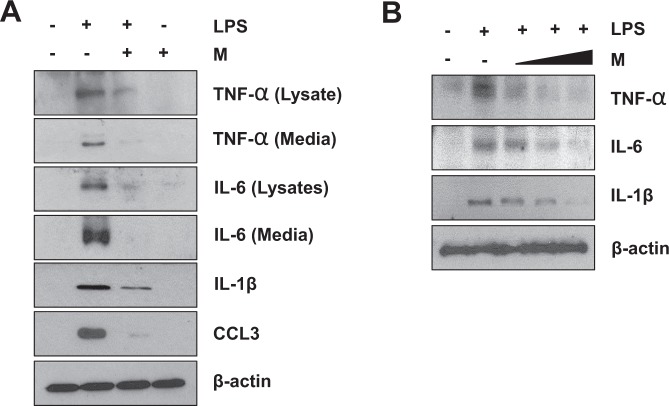
Menthol inhibits LPS-induced cytokine production in RAW 264.7 cells. (**A**) RAW 264.7 cells were pretreated with menthol, followed by stimulation with LPS for 6 hrs. Expression level of TNF-α, IL-6, IL-1β and CCL3 were induced by LPS and reduced in LPS+M compared to LPS. (**B**) Pretreatment of LPS (100 ng/mL) or control for 3 hrs and followed by induction with different concentration of menthol (50, 100 and 500 μmol/mL) for 6 hrs. Expression level of TNF-α, IL-6 and IL-1β were reduced by menthol treatment in a dose-dependent manner. β-actin was used as a loading control for western blot analysis.

The cytokine profile array results were independently tested again with western blot analysis. The RAW 264.7 cells used for this analysis underwent the same LPS and menthol treatment as the ones used for the cytokine arrays. Western blot data revealed that cells pre-treated with menthol had significantly reduced expression of TNF-α, CCL3, IL-6, and IL-1β (Fig. 4A). We next decided to use western blot analysis to examine if the effects of menthol were dose-dependent. RAW 264.7 cells were first pretreated with LPS (100 ng/ml) or control for 3 hrs. They were then incubated with varying concentrations of menthol (50, 100, 500 µmol/ml) for 6 hrs. Our results validated that menthol did indeed affect macrophages in a dose-dependent manner (Fig. 4B).

### Gene expression altered by menthol treatment

**Figure 5:**
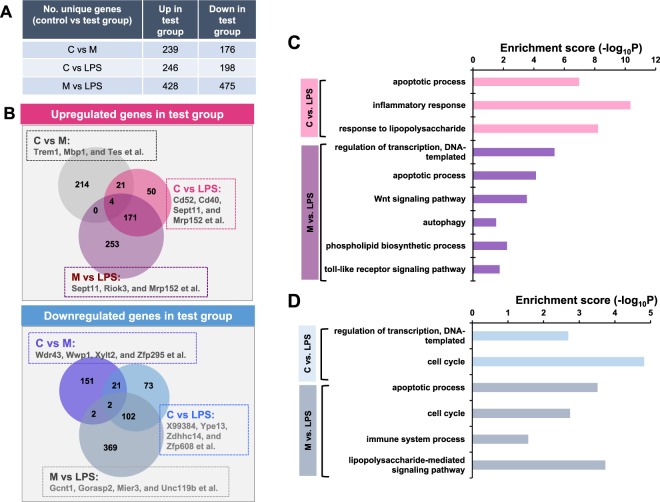
Differentially expressed genes in LPS, LPS+Menthol (LPS+M), and menthol (M) only conditions. (**A**) The number of DEGs perturbed by LPS or menthol treatment. (**B**) Venn diagram depicts shared and different DEGs. (**C,D**) Gene Ontology analysis suggested functional annotations (biological process) that were associated with up- (**C**) and downregulated (**D**) genes. Bar graph shows significantly enriched biological processes, which were up- and downregulated genes in test group. The inflammation events induced by LPS were inhibited by menthol in LPS mediated signaling pathway.

Further analysis using nucleotide microarrays validated our previous results showing that menthol mitigates LPS-induced inflammation in RAW 264.7 macrophage cells. Genes that were considered to be differentially expressed in C vs M, C vs LPS, and M vs LPS were selected, if they had a fold change >1.5 and p-value < 0.05. Figure 5A shows the number of upregulated and downregulated genes in each of the different groups. Figure 5B shows the list of DEGs for each group that were upregulated with menthol treatment; (C vs M) Trem1, MBP1, and TES; (C vs LPS) CD52, CD40, SEPT11, and MRP152; (M vs LPS) SEPT11, RIOK3, and MRP152. We also annotated downregulated DEGs for each group; (C vs M) WDR43, WWP1, and XYLT2; (C vs LPS) YPE13, ZDHHC14, and ZFP408; (M vs LPS) GCNT1, GORASP2, and MLER3.

To better understand the biological processes affected by menthol, we performed functional enrichment analyses based on GOBPs. DEGs were classified into different functional categories according to their GOBPs. Genes upregulated from LPS treatment were significantly associated with biological processes relating to inflammatory response (GO: 0006954) and response to lipopolysaccharides (GO: 0032496) (Fig. 5C). Genes downregulated by menthol treatment were those significantly involved in immune system processes (GO: 0002376) and the lipopolysaccharide-mediated signaling pathways of different groups (GO: 0031663) (Fig. 5D). These results indicate the LPS-driven inflammatory responses can be modulated by menthol treatment, suggesting that low levels of menthol may be associated with higher levels of inflammation from immune stimulants, such as LPS.

### Signaling pathways involved in the anti-inflammatory effects of menthol

**Figure 6:**
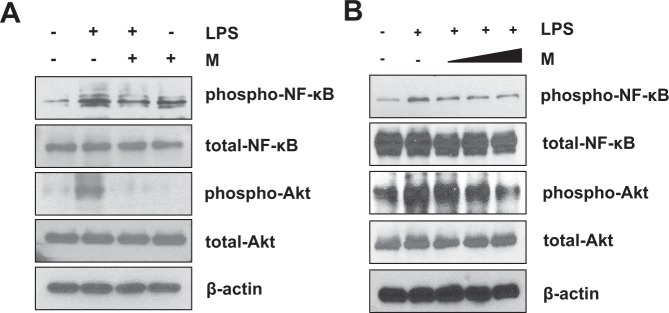
Menthol inhibits LPS-induced activation of NF-κB and Akt signaling pathways. (**A**) Phosphorylation levels of NF-κB and Akt were reduced by menthol treatment. (**B**) Phosphorylation of NF-κB and Akt were suppressed by menthol treatment in dose-dependent manner. β-actin was used as the loading control in western blot analysis.

We next wondered if the specific signaling pathways involved in LPS-stimulated cytokine perturbation can be modulated by menthol treatment. To determine the signaling pathways associated with the production and secretion of inflammatory cytokines, we screened the activation of key signaling pathways, including those of nuclear-factor-κB (NF-κB), Akt, and Erk1/2 MAPK. We found that the added presence of LPS induced phosphorylation of NF-κB and Akt in RAW 264.7 macrophage cells. Pretreatment with menthol significantly attenuated the phosphorylation levels of NF-κB and Akt, but not those of Erk1/2 MAPK (Fig. 6A, LPS+M compared to LPS). In addition, menthol treatment significantly reduced LPS-induced phosphorylation of NF-κB and Akt in a dose-dependent manner (Fig. 6B). This data suggest that menthol potentially inhibits LPS-induced inflammatory cytokines via the NF-κB and Akt signaling pathways.

### Nucleotide microarray analysis consistently showed that menthol affects production of LPS-induced cytokines in RAW 264.7 macrophage cells

**Figure 7:**
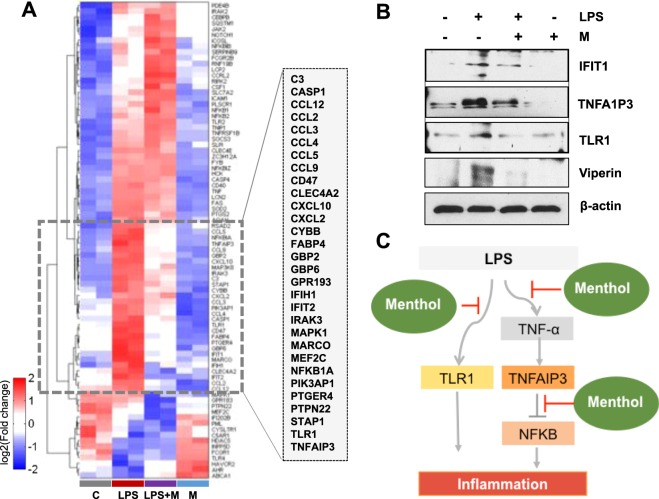
Menthol regulates the LPS-induced inflammatory response. (**A**) Heatmap image of DNA microarray data. RAW 264.7 cells were treated with LPS (100 ng/mL) with or without menthol (500 μmol/mL) for 6 hrs. The list of the genes in the enlarged box was sorted in order of gene symbol. (**B**) Cells were treated with LPS (100 ng/mL) with or without menthol (500 μmol/mL) for 6 hrs. Few candidates from (Figure A, Box): IFIT1, TNFA1P3, TLR1, and viperin were repressed by menthol treatment. (**C**) Hypothetical diagram showing how menthol may be attenuating the activation of the TLR1 and TNFAIP3-NF-κB signaling pathways; thereby, reducing downstream cytokine expression.

We next sought to identify DEGs in cells exposed to LPS with or without menthol via microarray analysis (Fig. 7A). Approximately 30% of the DEGs showed increased expression patterns when stimulated by LPS alone and decreased when treated additionally with menthol. Those DEGs included: C3, CCL2, CCL3, CCL4, CCL5, CCL9, CCL12, CXCL2, CXCL10, NFKB1A, toll-like microbial pattern recognition receptor 1 (TLR1), TNF-α induced protein 3 (TNFAIP3) *et al*. (Fig. 7A, box). Mapping of genes and protein expression from our study suggested that menthol suppresses the TLR pathway. The gene expression of interferon-induced protein with tetratricopeptide repeats 1 (IFIT1), TNFA1P3, TLR1, and viperin increased in response to LPS and decreased in response to menthol. To further validate the perturbed DEGs, western blot analysis was carried out. Protein levels of IFIT1, TNFA1P3, TLR1, and viperin changed consistent to gene expression changes (Fig. 7B).

Collectively, these experimental results propose a potential pathway through which TNF-α production and secretion is stimulated. This consequently leads to the activation of TLR1, TNFAIP3-NF-κB signaling, and inflammation of the bladder. These results also lead us to suggest that menthol could suppress IC-associated inflammation by blocking the activation of our hypothetical pathway (Fig. 7C).

## Discussion

Our GC-MS peak resolution and compound identification revealed unique urinary VOC profiles between IC patients and healthy controls. Based on these results, we further sought to understand the biological function of menthol, an identified IC-associated urinary metabolite. We identified this novel compound through recently improved data processing that could annotate unknown VOCs. These developments include database analysis tools, such as BinBase Database^21^, AMDIS^22^, SpectConnect (http://spectconnect.mit.edu), MZmine^23^, TagFinder^24^, MetAligh 3.0, and MetAlignID^25,26^. For this study, we used BinBase; this tool can be assessed by the public at http://vocBinBase.fiehnlab.ucdavis.edu.

### Cytokines and chemokines associated with IC

Although IC affects millions of people every year, the etiology of this disease remains elusive. The bladders of IC patients exhibit various pathophysiological alterations in the urothelium barrier lining, sensory nervous system, recruitment of immune cells, and activation of major signaling pathways. The inflammation that is associated with IC is potentially a result of inflammatory or bacterial agents. One such agent is LPS, a component of the bacterial outer membrane, which is known to bind to TLR4 and stimulate inflammation through upregulating the release of cytokines. Consequently, this increased production and secretion of cytokines leads to bladder inflammation, which induces an immune response and may be associated with additional urinary symptoms, such as hyper-excitability and pain^27,28^.

There have been a series of previous reports suggesting the use of inflammatory cytokines as IC biomarkers. They demonstrate that levels of cytokines, such as TNF-α, IL-2, IL-6, IL-8, and IL-1β, increase in the bladder and urine of IC patients. In our present study, we were able to observe elevated gene and protein expression levels of TNF-α, IL-6, IL-1β, and NF-κB in LPS-stimulated RAW 264.7 macrophage cells. TNF-α is a well-known pro-inflammatory cytokine that leads to the activation of inflammation, induces the expression of adhesion molecules, and contributes to the development of pain sensation. Prior studies have shown that pharmaceutical inhibition of TNF-α signaling or addition of neutralizing antibodies against TNF-α reduced the development of nociception in animal models. Collectively, the experimental data from our current study and preceding reports suggest that the hypersensitivity and/or inflammation seen in IC bladders may be treated by targeting inflammatory cytokines^28^.

### Menthol and IC-associated inflammation

Our VOC metabolomics analysis revealed that menthol expression is significantly less abundant in the urine of IC patients. Menthol, an aromatic and cyclic terpene alcohol, is a compound that is commonly used in a wide variety of products. Its main use is in relieving local inflammation, pain (e.g. joint aches), noxious heat, sensory hypersensitivity, sore throat *et al*. The analgesic and anti-inflammatory effects of menthol may be attributed to the fact that it is a TRPM8 agonist^29,30^. However, the underlying mechanisms of menthol’s biological effects remain obscure. Because of its mitigating effects, menthol was a particularly interesting compound in the context of IC. Chronic inflammation is typical in the pathogenesis of IC^31^, and higher levels of pro-inflammatory cytokines, such as macrophage-derived chemokines^32^ or urinary nerve growth factors, are reported to be associated with the disease^33,34^.

Given its anti-inflammatory effects, ability to suppress respiratory irritation, and association with pain relief, menthol is widely used for medicinal purposes^35,36^. Our study investigated the effectiveness of menthol on LPS-induced cytokine production and secretion. Interestingly, we found that levels of various inflammatory cytokines were suppressed by menthol. This was done through DNA microarrays and cytokine profiling; the results were verified through additional independent western blot analysis. We observed that menthol significantly reduced levels of TNF-α, IL-1β, IL-6, and CCL3 in LPS-stimulated RAW 264.7 macrophage cells. Previous literature has suggested that histamine release from mast cells is inhibited by menthol^37,38^. Furthermore, by regulating the NF-κB signaling pathway, menthol decreases carrageenan-induced inflammation processes^39^. Our results were also in accordance with these past findings and were similar to outcomes observed in menthol treatment of ulcerative colitis^40^ and ethanol-induced gastric ulcers^41^.

TNF-α is a pro-inflammatory cytokine involved in regulating a wide spectrum of biological processes, including cell differentiation, apoptosis, coagulation, and lipid metabolism. It is associated with a number of diseases, including autoimmune diseases, insulin resistance, and, most notably, cancer. NF-κB is protein complex responsible for DNA transcription, cytokine production, and basic survival in almost all animal cells^42^. Toll-like microbial pattern recognition receptors (TLRs) represent a non-self-recognition system that is hardwired to trigger inflammation^43^. In our present study, LPS-induced TLR1 was downregulated by menthol treatment. TNFAIP3, a protein whose expression is rapidly induced by TNF-α, is a ubiquitin-centered enzyme and has been shown to constrain NF-κB activation. The encoded protein is involved in most cytokine-mediated inflammatory responses; this is likely due to the presence of both ubiquitin ligase and deubiquitinase within the enzyme. Because of this, TNFAIP3 serves as a negative feedback regulator of NF-κB activation when TNF is present^44^. Our results provide new insights in LPS-induced inflammation, suggesting that it can be regulated by menthol via its suppression of TNF-α, TLR1, and/or TNFAIP3 and subsequent regulation of NF-κB activation. Altogether, this study indicates the potential placating role of menthol in IC patients.

Although further studies are warranted to ascertain the mechanistic basis of menthol’s observed anti-inflammatory effects, it would be interesting to investigate whether menthol can suppress any inflammation or sensory hypersensitivity in IC patients in the pre-clinical or clinical setting. Our discoveries provide potential therapeutic strategies; potent agonists of TNF-α, TLR1, TNFAIP3, and/or the NF-κB network may benefit IC patients through anti-hyperalgesic and anti-inflammatory effects.

### Accession numbers

The metabolomics data have been deposited in the Metabolomics Workbench Public Repository with the study ID number ST000603. The microarray data was deposited and is available at the Gene Expression Omnibus (http://www.ncbi.nlm.nih.gov/geo/), under the accession number GSE98933.

## Electronic supplementary material

Supplementary Figure 1
