# Supplementary material for: Menthol, a unique urinary volatile compound, is associated with chronic inflammation in interstitial cystitis
Source: Sci Rep. 2018 Jul 18;8:10859. doi: 10.1038/s41598-018-29085-3 (PMC6052149; doi:10.1038/s41598-018-29085-3)

Menthol, a unique urinary volatile compound, is associated with chronic inflammation in interstitial cystitis

Muhammad Shahid[1], Min Young Lee[2], Austin Yeon[1], Eunho Cho[3], Vikram Sairam[3], Luis Valdiviez[4], Sungyong You[1], Jayoung Kim [1,3,5,6,¶]

[1]Departments of Surgery and Biomedical Sciences, Cedars-Sinai Medical Center, Los Angeles, CA, USA; [2]Institute for Systems Biology, Seattle, WA, USA; [3]University of California Los Angeles, CA, USA; [4]West Coast Metabolomics Center, UC Davis, Davis CA 95616; [5]Samuel Oschin Comprehensive Cancer Institute, Cedars-Sinai Medical Center, Los Angeles, CA, USA; [6]Department of Urology, Ga Cheon University College of Medicine, Incheon, South Korea

[¶]**Correspondence:**
Jayoung Kim, PhD.
Departments of Surgery and Biomedical Sciences, Cedars-Sinai Medical Center, 8700 Beverly Blvd., Los Angeles, CA 90048
Tel: +1-310-423-7168
Fax:+1-310-967-3809
E-mail: Jayoung.Kim@cshs.org

Supplementary Figure Legends

Supplementary Figure 1. (A) Histogram displays distribution of levels of metabolite intensities after normalization by quantile method. **(B)** Heatmap shows correlation matrix of metabolite profiles. Red and blue indicate positive and negative correlation, respectively.

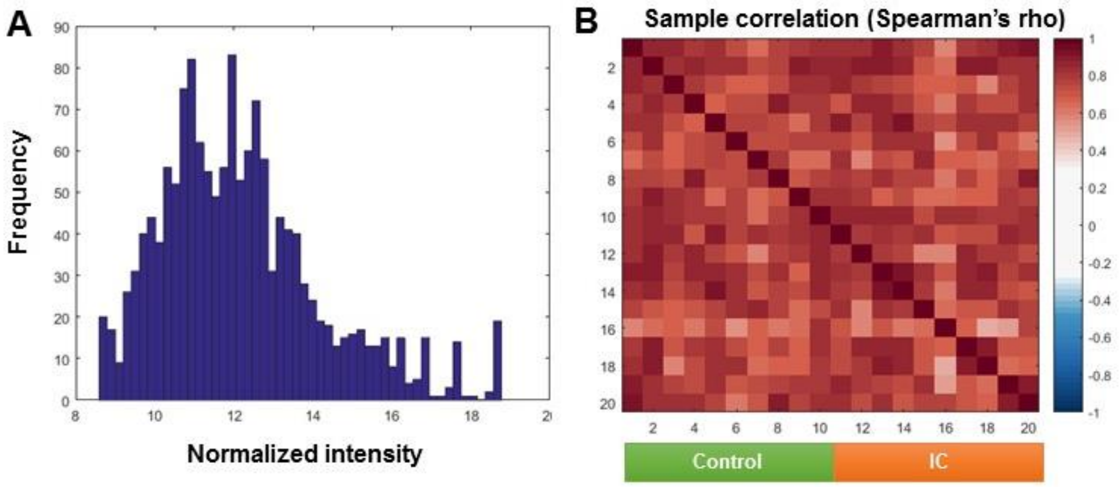

Supplement: Supplementary file 1 — Supplementary Figure 1 [file 41598_2018_29085_MOESM1_ESM.pdf]
